# Accelerometer-Measured Physical Activity in Children and Adolescents at Altitudes over 3500 Meters: A Cross-Sectional Study in Tibet

**DOI:** 10.3390/ijerph16050686

**Published:** 2019-02-26

**Authors:** Ming-jian Nie, Chao-qun Fan, Rui-zhe Sun, Jing-jing Wang, Qiang Feng, Yan-feng Zhang, Zhi Yao, Mei Wang

**Affiliations:** 1 National Physical Fitness Research Center, China Institute of Sport Science, Beijing 100061, China; nie.mj@outlook.com (M.-j.N.); chaoqunfan@126.com (C.-q.F.); wangjingjing@ciss.cn (J.-j.W.); fengqiang@ciss.cn (Q.F.); zhangyanfeng@ciss.cn (Y.-f.Z.); 2 Tibet Institute of Sport Science, Lhasa 850007, China; supersun.123@163.com (R.-z.S.); yz2582598350@163.com (Z.Y.)

**Keywords:** physical activity, children, adolescents, plateau, accelerometer, Tibet

## Abstract

There is a scarcity of studies on the physical activity (PA) of children and adolescents who live at high altitudes. This study aimed to objectively assess PA of children and adolescents living in the Tibet at altitudes over 3500 m and to examine its difference by ethnicity, gender, age/grade, and body weight status groups. A sample of 397 students aged 9–18 years were recruited from 7 schools in Lhasa, Tibet. PA was measured using accelerometers (ActiGraph GT3X) for seven consecutive days and moderate to vigorous PA (MVPA) was identified using the Evenson (2008) cut-points. Participant MVPA was 62.3 min/day, with 65.5 min/day during weekdays and 54.1 min/day on weekends. Indigenous Tibetans were more active than Hans, and boys had more MVPA than girls. Age had a significantly weak negative correlation with MVPA. There was no significant difference in MVPA between the non-overweight and overweight/obese groups. Overall, only 9.1% (13.8% in boys and 4.5% in girls) accumulated at least 60 min of MVPA per day. Compared to their counterparts in other regions, the daily MVPA of children and adolescents living on the Tibetan Plateau at altitudes over 3500 m was relatively high. However, the proportion of meeting the WHO’s PA recommendations was extremely low.

## 1. Introduction

Regular physical activity (PA) has been demonstrated to affect weight control [1], musculoskeletal health, cardiovascular risks [2], motor skill development [3], cognitive ability [4], academic performance [5,6], and self-esteem [7]. PA is also associated with a reduction in all-cause mortality [8]. The evolution of the human body is such that most systems do not develop and function in the best way unless stimulated by frequent PA, including the bones, muscles, metabolism, and cardiovascular systems [9]. Therefore, a lack of PA can lead to many health risks, and in 2009 it was identified by the World Health Organization (WHO) as the fourth leading risk factor for worldwide deaths (6% of global deaths) [10].

PA is the foundation of health for children and adolescents, who are at critical stages of physical and mental development [11]. A dose-response analysis on PA and health suggested that a cumulative 60 minutes (min) of moderate to vigorous physical activity (MVPA) per day has important health benefits for most children and adolescents [12,13]. The WHO recommends that children and adolescents should accumulate at least 60 min/day of MVPA to obtain the necessary health benefits [11]. Governments have issued many policies to encourage PA among children and adolescents to meet this recommendation, but the proportion of this population with insufficient PA is still increasing worldwide. Four-fifths of the world’s adolescents do not meet the WHO’s recommended level [14].

The lack of PA among children and adolescents is severe in China as well. In 2014, the results of a survey on Sports and Physical Fitness among Chinese people aged 6-69 years in 2014 showed that only 8.9% of children and adolescents met the WHO recommendations [15]. In 2016, the “PA and Fitness in China—The Youth Study” (PAFCTYS) survey showed that the self-reported average daily MVPA for children aged 9-17 years was 45.4 min (boys: 47.2 min/day, girls: 43.7 min/day), and about 30% of participants met the MVPA recommendations [16]. Another large-scale survey used accelerometers to assess PA among students and found that Chinese urban children and adolescents spent an average of 28.26 min/day doing MVPA, and only 9.4% of boys and 1.9% of girls met the recommendation of 60 min/day of MVPA [17]. 

China’s Qinghai-Tibet Plateau is the largest plateau (2.5 million km^2^) with the highest altitude (>4000 m) and has a relatively high population (>12 million) [18]. Indigenous Tibetans are the major aborigines of the Qinghai-Tibet Plateau. They settled the Qinghai-Tibet Plateau as early as 30,000 years ago and began to develop physiological adaptations to the low-oxygen environment [19]. A large number of lowland people (mainly Han people) have also migrated to the plateau with the Chinese western development strategy and “Go West” campaigns. The Sixth National Population Census of China (2010) showed that the Indigenous Tibetan and Han populations respectively account for 90.48% and 8.17% of the population of Tibet and comprise the main ethnic groups of the Qinghai-Tibet Plateau [20]. Moreover, according to the National Bureau of Statistics of China, the regional Gross Domestic Product (GDP) and the per capita disposable income of Tibet ranked last among the 31 provinces from 2013 to 2017 in China [21]. 

The characteristics of low oxygen, low temperature, strong ultraviolet radiation, and large climate differences make the regional environment between Tibet and inland China very different. It is well known that living at altitudes above 3000 m can have an impact on the human body [22,23]. As a result, congenital heart disease, hypertension, and other diseases have high morbidity in Tibet and may be influenced by the extreme living environments [24]. The plateau environment not only impacts the human body but also lifestyles, and thus may also affect PA. Several studies have reported on the growth and development characteristics of children and adolescents in Tibet [25,26], as well as the related situations of eye diseases [24], rickets/Kaschin-Beck disease [27,28], and nutrition [29,30,31]. However, no study has examined PA. Moreover, to our knowledge, there is a lack of research on PA in children and adolescents living on high altitude plateaus worldwide. 

Understanding the levels and characteristics of PA among native Tibetan children and adolescents and among lowland migrants on the plateau is of great significance for promoting behavioral health, improving PA, and improving health status. Therefore, this study objectively assessed the PA of children and adolescents living in the Tibetan Plateau by using accelerometers. Furthermore, the differences in PA were compared with respect to ethnicity, gender, age/grade, and weight status groups.

## 2. Materials and Methods 

### 2.1. Design and Participants 

**Figure 1:**
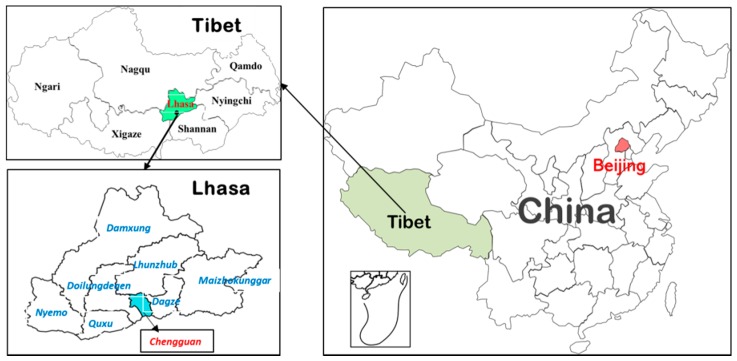
Map of Investigation on Physical Fitness and Health Behaviors of Tibetan School-Age Children and Adolescents (IPFHB-TSCA) research site.

This study is part of the "Investigation on Physical Fitness and Health Behaviors of Tibetan School-Age Children and Adolescents (IPFHB-TSCA)." The IPFHB-TSCA is a cross-sectional study conducted by the Tibet Institute of Sport Science (TISS) and the China Institute of Sport Science (CISS) to explore the physical fitness, growth development, and health behaviors of children and adolescents on the plateau. There is a special geographical environment in Tibet with low population density. However, the schools have a concentrated distribution. According to educational organization statistics, 33 out of 94 schools are located in the Chengguan District (CGD) of the city of Lhasa (out of a total 8 districts in the city). Over half of the junior high schools and two-thirds of the senior high schools are in CGD. Therefore, we chose CGD as the sampling site for IPFHB-TSCA (Figure 1). Three elementary schools, two junior high schools, and two senior high schools in CGD were randomly selected. Data were collected from June to October 2017. The study was approved by the Ethics Committee of CISS (Ethical code: CISSIRD-2016006).

The participants were in 4th to 12th grade and were recruited from seven schools. Students were excluded if they answered “yes” to any question on the Physical Activity Preparation Questionnaire (PAR-Q) that indicated it would be unsafe for them to perform moderate physical activity (MPA) [32]. More than 2000 students aged 9–18 years were included in the IPFHB-TSCA, of which 79.4% were indigenous Tibetans, and the remaining were lowland immigrants or descendants represented by the Han ethnicity. Prior to the data collection, all subjects signed an informed consent form and were told that they could refuse to participate in the test or answer any questions and withdraw at any time. 

A sub-sample of the participants was selected from the IPFHB-TSCA study. Quota sampling was conducted with the ratios of genders and ethnicities at 1:1. Forty-eight students were randomly selected from each grade, and 480 subjects were finally included to wear accelerometers. Subjects were divided into three according to their grade level: primary school (PS), junior high school (JHS), and senior high school (SHS). 

### 2.2. Procedure 

A pilot study of the IPFHB-TSCA was conducted in June 2017 in the CGD of Lhasa City. The results showed that all instruments and equipment can operate normally on the plateau, including accelerometers. The questionnaire could be understood well by indigenous Tibetan and Han students. The main study was conducted from August to September 2017. The research team provided intensive training on the procedure of the study, questionnaire items, and instruments used for postgraduate students who would help to collect data. 

### 2.3. Measures

The participants involved in the IPFHB-TSCA received tests on physical fitness-related outcomes and questionnaires. Based on the objectives of the current study, only demographic characteristics, body mass index (BMI), and PA data were reported. 

#### 2.3.1. Demographic Characteristics

Demographic data, including ethnicity, gender, boarding status, residential location, and birthdate, were obtained with questionnaires. The main framework of the questionnaire is based on the localization revision of the WHO’s Health Behavior in School-Aged Children (HBSC) study [33]. The questionnaires could be used well in Lhasa primary and secondary schools according to the pilot study. 

#### 2.3.2. Height, Weight, and Body Mass Index (BMI)

Height and weight were measured using a validated electronic height and weight scale (Jianmin Brand, Beijing, China) according to a standard protocol. Height was accurate to 0.1 cm, and weight was accurate to 0.1 kg. BMI was calculated as the weight in kilograms divided by the square of the height in meters. Subjects’ weight statuses were determined according to the BMI reference norm for screening overweight and obesity cut-points for Chinese children and adolescents issued by the Working Group of Obesity, China (WGOC), International Life Science Association [34]. The subjects were divided into non-overweight (Non-OW) and overweight/obesity (OW/OB) groups.

#### 2.3.3. Physical Activity 

PA was objectively assessed using ActiGraph GT3X accelerometers (ActiGraph, Fort Walton Beach, FL, USA), which have shown high reliability and validity among children [35]. It has also been confirmed that the device can be operated normally at high altitudes [36]. Participants were asked to wear the accelerometers for 24 h a day for 7 consecutive days on the right hip. The accelerometers were secured by an elastic belt. The accelerometer could only be removed during water-related activities (e.g., swimming, showering, and bathing). Epochs of 5 s [37] and a sampling frequency of 60 Hz were adopted [38,39]. In addition, Monday to Friday were school days for participants, and Saturday to Sunday were rest days in this study.

The accelerometer data were downloaded onto a computer using the software ActiLife 6.13.3. For analysis, the extreme values (>20,000 counts per minute (CPM)) were removed, and non-wear time was defined as 20 min or more of consecutive zeros [40]. A valid day was defined as having 10 h or more of wear time [41]. The accelerometer data were included in the final analysis if they contained at least three valid weekdays and one valid weekend [42]. Based on recent recommendations [43], the cut-points reported by Evenson (2008) were used to classify PA with units of CPM as sedentary (0–100 CPM), light PA (LPA) (101–2295 CPM), moderate PA (MPA) (2296–4011 CPM), vigorous PA (VPA) (≥4012 CPM), and MVPA (≥2296 CPM).

### 2.4. Statistical Analysis 

Data were analyzed using JMP statistical software version 13.2.0 (SAS Institute Inc., Cary, NC, USA). Results were presented as numbers and percentage for categorical data and as mean and standard deviation for continuous data, respectively. Shapiro–Wilk test was performed to test data distribution. The Wilcoxon two-sample test was used to test inter-group differences in MVPA with respect to ethnicity, gender, and body weight group, and the differences in MVPA between grade groups were assessed by using the Steel–Dwass test. The Wilcoxon Matched-Pairs Signed Ranks Test was used to test the differences in MVPA between weekends and weekdays. Percentage values of inter-group differences were tested by the chi-squared test. Partial correlation was used to analyze the trend of MVPA with age. Statistical significance was set at *p* < 0.05.

## 3. Results

### 3.1. Characteristics of the Participants

The participants’ characteristics are presented in Table 1. Of the 480 participants, five were excluded due to loss or damage of the accelerometer. Of the 475 remaining participants, 397 (82.7%) provided valid accelerometer data. The average wearing time of the subjects was 894.8 ± 56.1 min/day (14.9 h), which was much longer than the minimum required effective wearing time of 600 min/day (10 h) that was set as the filter standard. 

### 3.2. PA Levels 

Table 2 shows the participants’ PA levels. The average MVPA time reached 62.3 min/day (SD = 21.7), with 65.5 min/day (SD = 22.3) during weekdays and 54.1 min/day (SD = 32.2) on weekends. Subjects had significantly higher MVPA during weekdays than on weekends (*p* < 0.001), regardless of ethnicity, gender, and grade groups (Table 3).

### 3.3. Meeting PA Guidelines

Table 3 shows the percentage of participants who had 60 min/day of MVPA for every day or over five days per week. Overall, only 9.1% of the subjects were able to accumulate at least 60 min/day of MVPA. Nearly three times as many indigenous Tibetans met the WHO’s recommendation as Hans (13.5% vs. 4.6%, x^2^ = 9.601, *p* = 0.0019). The analysis of gender groups showed that 13.8% of boys accumulated at least 60 min/day of MVPA, but this was observed in only 4.5% of girls (x^2^ = 10.611, *p* = 0.0011). Moreover 10.1%, 9.7%, and 7.1% of PS, JHS, and SHS students reached the WHO’s recommendation, respectively. No group difference was found in the rates of meeting the WHO recommendation between the Non-OW (9.0%) and OW/OB (9.8%) groups.

To explore the subjects’ PA further, we also examined whether the students were able to achieve 60 min/day of MVPA or more for at least five days per week. Compared to the rate of daily MVPA, the percentage of participants who had 60 min/day of MVPA for over five days per week increased to 34.8%. There was a significant difference between indigenous Tibetans and Hans in the percentage of participants achieving 60 min/day of MVPA over five days per week (41.5% vs. 27.9%, x^2^ = 8.072, *p* = 0.0045). Similarly, the rate of boys was significantly more than girls (51.3% vs. 18.8%, x^2^ = 46.128, *p* < 0.001). Furthermore 35.3%, 37.9%, and 30.1% of PS, JHS, and SHS students achieved this amount of MVPA, respectively. No group difference was found between the Non-OW (33.7%) and OW/OB (43.9%) groups.

### 3.4. Daily Changes in PA Levels

**Figure 2:**
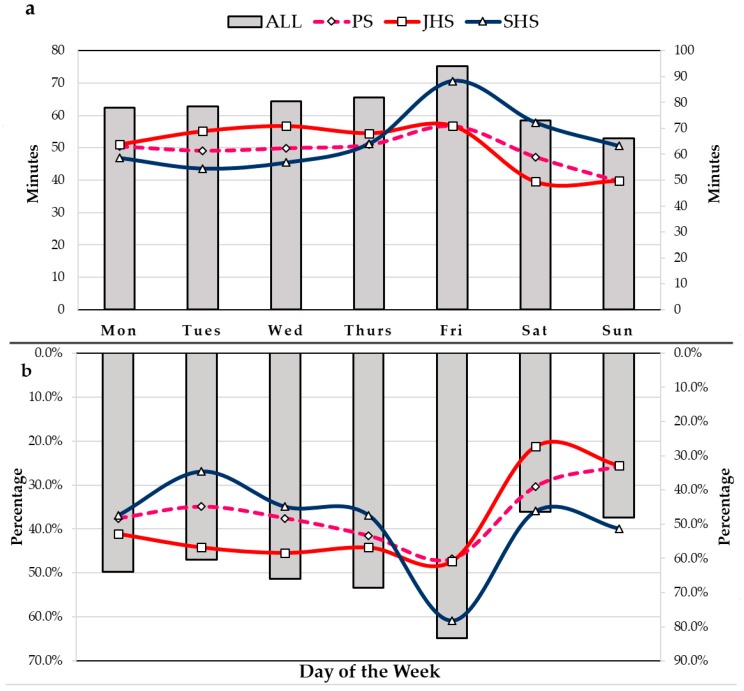
The daily changes in PA levels from Monday to Sunday (**a**,**b**). Notes: MVPA = Moderate to Vigorous Physical Activity; MVPA% = The proportion of participants who had MVPA for more than 60 min each day; Mon = Monday; Tues = Tuesday; Wed = Wednesday; Thurs = Thursday; Fri = Friday; Sat = Saturday; Sun = Sunday; PS = Primary School; JHS = Junior High School; SHS = Senior High School; a Trends of average daily MVPA time in PS, JHS, SHS and all students from Monday to Sunday; b Trends of the proportion of participants who had MVPA for more than 60 min each day in PS, JHS, SHS and all students from Monday to Sunday; Data were obtained from 321 subjects who worn the accelerometer for 7 valid days.

Figure 2 shows the participants’ MVPA in a week from Monday to Sunday. Students’ MVPA increased in the weekdays and decreased significantly on Saturday and Sunday (Figure 2a). The similar trend was seen for the proportion of participants who had MVPA for more than 60 min each day (Figure 2b). From Monday to Thursday, students in the JHS group had the highest PA levels, while the SHS group had the lowest. The opposite is true from Friday to Sunday, where the SHS group had the highest PA level, while the JHS group had the lowest.

### 3.5. PA Differences in Ethnicity, Gender, Age/Grade, and Weight Status

Table 3 also shows the differences in MVPA in terms of ethnicity, gender, age/grade, and weight status. Indigenous Tibetans (67.4 ± 22.7 min/day) had significantly higher MVPA per day than Hans (57.2 ± 19.5 min/day) (*p* < 0.001) on both weekends (*p* < 0.0001) and weekdays (*p* = 0.0085). The ethnic differences in MVPA on weekends were more pronounced than on weekdays (*p* < 0.0001 vs. *p* = 0.0085). Boys accumulated an average of 71.5 (SD = 22.3) min/day of MVPA, which was significantly higher than girls, who accumulated 53.5 (SD = 17.0) min/day (*p* < 0.001). Boys’ daily MVPA was significantly higher than that of girls on both weekends (*p* < 0.001) and weekdays (*p* < 0.001).

**Figure 3:**
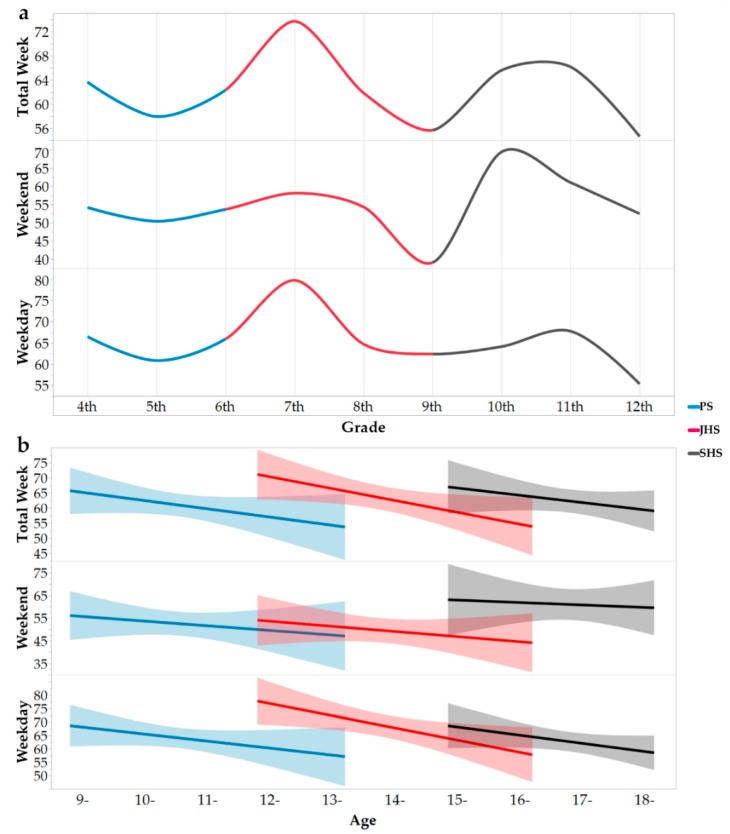
The changing trend of MVPA with grade/age (**a**,**b**). Notes: MVPA = Moderate to Vigorous Physical Activity; PS = Primary School; JHS = Junior High School; SHS = Senior High School; a The changing trend of MVPA with grade; b The fitting trend of age and MVPA in different grade groups.

The levels of MVPA achieved by the students from the PS, JHS, and SHS groups were 61.5 (SD = 21.8) min/day, 63.5 (SD = 22.8) min/day, and 61.9 (SD = 20.4) min/day. There was no significant difference between the three school groups of MVPA on both weekends and weekdays. After controlling for ethnicity, gender, boarding status, residential location, and wear time, the partial correlation analysis showed that age had a significantly weak negative correlation with MVPA time (r = −0.241, *p* < 0.001) on both weekends (r = −0.165, *p* < 0.01) and weekdays (r = −0.214, *p* < 0.001). Moreover, the MVPA of students entering the first year of the higher learning stage was significantly higher than that of the students who remained in the last year of the lower learning stage, that is, the MVPA of the students entering the new learning stage (e.g., from the 6th to 7th grade, from the 9th to 10th) increased in a temporary period (Figure 3a). However, within the same grade group, MVPA always tends to decline with age (Figure 3b). 

In addition, the difference of daily MVPA between the Non-OW group and the OW/OB group was not statistically significant on both weekdays and weekends.

## 4. Discussion

This study is the first to report the PA in children and adolescents living in highland areas (≥3500 m). The findings showed that children and adolescents living on the Tibetan Plateau accumulated MVPA at 62.3 min/day, but only 9.1% met the WHO’s recommendation (13.8% of boys and 4.5% of girls). Furthermore, ethnicity, gender, and age/grade were identified as factors associated with the students’ MVPA. Indigenous Tibetans had higher MVPA than Hans, and boys were more active than girls. However, MVPA did not differ significantly between the three school groups, and the level of MVPA of the students would temporarily increase at entrance to the higher learning stage. However, there was still a tendency for MVPA to decline with age, especially within the same grade group. We also found that MVPA was significantly higher on weekdays than weekends. No significant difference in MVPA was observed between the Non-OW and OW/OB groups. 

The comparison of accelerometer-based findings across studies should be interpreted with caution, considering that large variations can result from the different methods used in different studies, including the definition of valid wear time, number of days, and cut-points adopted to categorize the level of PA [44]. However, we found several studies with the same cut-points standards of MVPA in different ethnic populations. The average daily MVPA of children and adolescents in this study (62.3 min/day) was lower than that reported for children and adolescents in the Senegal (75.0 min/day) [45]. However, the present result was slightly higher than that in the UK (60.5 min/day) [46], Tunisia (59.8 min/day) [47], Malta (50.0 min/day) [48], the USA (48.0 min/day) [49], and Finland (49.2 min/day) [50], as well as significantly higher than that in Australia (36.6 min/day) [51]. Another survey of Chinese students living in plain urban cities reported that the average MVPA was 28.26 min/day, which is much lower than that observed for Highland Chinese children and adolescents in this study [17]. This difference could be largely attributed to the different cut-points used in these two studies (2296 CPM in this study vs. 2800 CPM in the other study), in addition to the difference in region and altitude.

The unique conditions of the plateau environment include low air pressure, low oxygen levels, dryness, cold weather, intense solar infrared, and ultraviolet radiation, etc. [24]. These characteristics are inherently detrimental to human survival since they affect the human body and lifestyle. However, the results of the comparison revealed that the children and adolescents on the Tibetan Plateau accumulated a relatively high amount of daily MVPA, which may suggest that the impact of the plateau environment on the PA of children and adolescents does not seem to be as serious as believed. Due to the lack of data on PA in regions with high altitude, our research can only be compared with results for lowland populations. Future research on the influence of different elevations on PA is required.

An interesting finding is the relatively high MVPA and the low rate of meeting the WHO guideline. Only 9.1% of the participants accumulated 60 min of daily MVPA. This is substantially lower than in Malta (24.7%) [48], the USA (24.3%) [49], Australia (22.1%) [51], Senegal (54.8%) [45], and Tunisia (47.5%) [47]. In the HBSC study, children with a daily MVPA of at least 60 min for 5 or more days were considered to be physically active [52]. According to this classification, we found that 34.8% of participants were active. We assumed that children and adolescents living on the Tibetan Plateau may be active on several specific days of the week rather than being active daily throughout the whole week. This assumption could be explained by the MVPA results for each day. MVPA was significantly higher on weekdays than on weekends (*p* < 0.001. Nearly half of the participants achieved at least 60 min of MVPA every day from Monday to Friday, but this level showed a precipitous decline on weekends and fell to a minimum of 36.1% by Saturday. Therefore, the reduction of MVPA over the weekend may cause a very low result of PA for children and adolescents living on the Tibetan Plateau.

Consistently, the decline in PA on weekends also occurs in other countries and regions [53,54]. For example, Jurak et al. found that the PA of children and adolescents in three different cities decreased from school days to the weekend, and the MVPA of girls fell by 11 min to 66 min [55]. The reason for the decline in MVPA on weekends among Chinese subjects may be related to the educational environment. In traditional Chinese culture, adequate PA among children is not a priority for parents. Families and society pay too much attention to academic courses in hopes that their children will achieve top grades. This mentality is quite universal in Chinese culture [56]. Therefore, children have to spend most of their spare time learning and have less time to exercise [15]. These circumstances may be more serious on weekends since there is no compulsory physical education class or exercise. Furthermore, weekends are occupied by a large amount of sedentary behavior time due to various extracurricular tutoring.

The study also showed that the indigenous Tibetans were more active than the Hans. This finding is consistent with the only two other studies that compared PA between indigenous Tibetan and Han students in China. Du et al. [57] and Zhang et al. [58] found through a one-year retrospective questionnaire that the overall weekly PA of indigenous Tibetan students was significantly higher than that of Han students (*p* < 0.05). 

As an indigenous group, Tibetans have the best physiological adaptability to the low-oxygen environment of the plateau [59,60]. For example, in terms of morphology, indigenous Tibetans have a distinct chest depth among boys and chest width among girls [25,61]. Physiologically, the typical adaptation characteristics of Tibetans to living on the plateau are higher ventilation, lower pulmonary arterial pressure, higher blood oxygen saturation, and relatively low hemoglobin concentrations [60,62]. The Hans did not begin moving to Tibet on a large scale until after the 1970s [63] and have not fully established adaptation mechanisms to the plateau environment because of the short migration time. Although the impact of high-altitude living on athletic ability is not entirely clear, this effect is more pronounced in immigrant lowland populations than in local Tibetans [64]. Previous studies have also found that indigenous Tibetan teenagers have better athletic abilities than Han immigrants [65]. Genetic and physiological adaptation mechanisms for hypoxia could be the reasons for indigenous Tibetans having higher PA than Hans. 

In addition, lifestyle differences resulting from cultural differences may also have affected the results. This is also a possible reason why the differences in PA between indigenous Tibetans and Hans were mainly reflected on the weekends. On the weekend, children and adolescents return to the cultural and living environments of their individual ethnicities instead of uniformly doing the same activities organized by the school on weekdays. In the routine life of Tibetans, many religious activities are moderately to intensely active, such as turning galleries, mountain climbing, prayer wheels, and long kowtows [66], as well as dances, horse racing, tug-of-war, and wrestling [67]. These factors could also contribute to indigenous Tibetans’ high levels of PA.

In line with other studies, this study also found a gender difference in PA. Globally, boys generally have higher PA than girls, which has been observed using self-reports and objective measurements [14,68,69]. The gender differences in PA may be related to several aspects of participation in physical exercise between boys and girls. Girls may have more restrictions with respect to accessibility, time, peers, psychology, and knowledge of PA than boys [70], as well as lower self-confidence and self-esteem in PA and sports activities [71]. For safety reasons, parents might allow boys to have more outdoor exercise than girls [72]. Moreover, interest in exercise is lower among girls than boys [73], and the expectation to participate in physical exercise is significantly higher for boys than girls [74]. Boys are also more likely to engage in high-intensity and confrontational sports, such as basketball and football, while girls are less interested and willing to take part in these activities [75].

A retrospective study from 1993 reported that the PA of school-age boys fell by 2.7% per year, while the rate of decline in girls per year was as high as 7.4% [76]. Since then, other scholars have also reported similar findings that PA declines with the increase of age [77,78,79]. In the present study, PA also showed a trend of decreasing with age. The comparison of the one-week changes in PA levels in the PS, JHS, and SHS groups and the absence of significant differences between the three school groups of MVPA showed that this decline was not consecutive. This was because as the students enter the higher learning stage, MVPA temporarily increased, and in the same grade group, it tended to decline with age. Therefore, partial correlation analysis showed a significantly weakly negative correlation between MVPA and age (r = −0.241, *p* < 0.001). This may be related to the periodic change of academic pressure. After the entrance examination, academic pressure would be temporarily reduced, but with higher grades, the learning pressure may elevate again, which may result in a decline of MVPA. However, in view of the fact that the present study is a cross-sectional survey, the true situation and causes of the change in PA with age/grade in this population remains to be confirmed by further longitudinal follow-up studies, which will help to determine the key time nodes of PA intervention.

Over the past three decades, overweight and obesity among children and adolescents has become a worldwide problem [80] and has brought about a wide range of social, psychological, and medical burdens [81]. PA has been considered as a basis for maintaining energy balance and weight control [11]. The lack of PA is considered to be an important factor in the occurrence and development of overweight or obesity in children and adolescents [82]. However, the results of the study did not find the relationship between PA and weight status. Some studies have found a negative correlation between PA and BMI [83,84], and the risk of overweight or obesity also decreases with the increase in the number of days with 60 min of MVPA among children and adolescents [85]. However, a systematic review by Annette et al. showed that 7 out of 11 studies found no relationship between PA and overweight [86]. The insignificant association may suggest that more rigorous trials should be implemented to examine the association of PA with overweight or obesity in children and adolescents in order to eliminate interference from other confounding factors that are more closely related to BMI, such as dietary factors.

The main advantage of this research is that it is the first study to evaluate the PA of children and adolescents on the Tibetan Plateau using an objective measuring device. It is also the first time in the world to reveal the PA of children and adolescents living at an altitude of ≥3500 m. This study supplements the data from plateau regions among international studies on PA internationally. In addition, the subjects in this study were required to wear accelerometers for 24 h, so the collected data could reflect the daily PA of children and adolescents more accurately, which could greatly improve the effective wearing time of the samples.

This study also had some limitations. First, the study was conducted in only Lhasa and used quota sampling rather than random sampling, although the latter method is not applicable to all of Tibet. However, considering the special geographical environment of Tibet, the dense geographical distribution of schools at all levels, and the difficulty of obtaining Han samples, the samples are still very representative. Second, the data were obtained from June to September, which is the most pleasant season in Tibet throughout the year. Therefore, the results may reflect the highest level of PA among children and adolescents living on the Tibet during a year. Third, accelerometers do not record the type of PA, which prevents us from exploring the frequency of specific behaviors and interpreting the observed differences in PA between ethnicity, gender, and grade groups [69]. Nevertheless, accelerometers are considered to provide good objective measures of PA because they minimize self-reporting bias and eliminate human error when recalling PA [87]. Finally, this study is a cross-sectional survey. The trend of change in PA with respect to age/grade needs to be verified by longitudinal or cohort studies.

## 5. Conclusions

Compared to other regions, the daily MVPA of children and adolescents living on the Tibetan Plateau was relatively high. However, the significant reduction in PA over the weekend resulted in an extremely low rate of meeting the WHO’s PA recommendation. MVPA difference between the indigenous Tibetans and Hans mainly occurred on the weekend, so the policies and strategies aimed at increasing PA should focus on the weekends to increase the level of PA and reduce its ethnic differences. The trend of PA with age deserves further research and confirmation, especially before and after entering the new learning stage, which will help determine the important time nodes of the PA intervention strategy. Indigenous Tibetans had higher MVPA than Hans, and boys were more active than girls, but no significant differences in PA were found in different BMI categories. In summary, our research has provided the first account of objectively measured PA among a sample of children and adolescents living on the Tibetan Plateau. As an observational study, we cannot verify or explain the causes of this phenomenon. However, researchers and policy-makers can use this information to provide reference and inspiration for their future work.

## Figures and Tables

**Table 1:** Description of the study sample.

Study Variables	Sample size *n* (%)	Age (years) M (SD)	Height (cm) M (SD)	Weight (kg) M (SD)	BMI (kg/m^2^) M (SD)
Overall	397 (100%)	13.5 (2.7)	154.3 (12.9)	45.4 (13.4)	18.7 (3.3)
Race					
Tibetan	200 (50.4%)	13.6 (2.8)	154.9 (12.0)	46.4 (12.8)	18.9 (3.2)
Han	197 (49.6%)	13.3 (2.7)	153.6 (13.7)	44.4 (14.0)	18.4 (3.3)
Gender					
Male	195 (49.1%)	13.5 (2.8)	156.9 (15.1)	47.0 (15.3)	18.5 (3.3)
Female	202 (50.9%)	13.4 (2.7)	151.7 (9.6) ^a (***)^	43.8 (11.1)	18.8 (3.2)
Grade by School Level					
PS	139 (35.0%)	10.5 (1.0)	141.8 (8.5)	32.6 (7.3)	16.1 (2.1)
JHS	145 (36.5%)	13.7 (1.3)	158.3 (8.5)	48.9 (9.1)	19.4 (2.6)
SHS	113 (28.5%)	16.8 (0.9)	164.4 (9.1)	56.8 (10.7)	20.9 (3.2)
Weight Status					
Non-OW	356 (89.7%)	13.3 (2.7)	153.6 (12.9)	43.2 (11.6)	17.9 (2.4)
OW/OB	41 (10.3%)	14.5 (2.6) ^b (*)^	160.4 (10.7) ^b (**)^	64.6 (13.0) ^b (***)^	24.9 (3.1) ^b (***)^

Notes: M = Mean; SD = Standard Deviation; BMI = Body Mass Index; PS = Primary School; JHS = Junior High School; SHS = Senior High School; Non-OW = Non-overweight; OW/OB = Overweight/Obesity; ^a^ Significant difference (*p* < 0.05) between Male and Female; ^b^ Significant difference (*p* < 0.05) between Non-OW and OW/OB; * *p* < 0.05, ** *p* < 0.01, *** *p* < 0.001.

**Table 2:** PA time for each intensity of participants for total week, weekend and weekday.

PA (Min/Day)	Whole Week M (SD)	Weekend M (SD)	Weekdays M (SD)
LPA	207.9 (48.5)	185.0 (52.8)	216.7 (53.7) ^φ (***)^
MPA	43.0 (13.4)	37.5 (21.0)	45.1 (14.0) ^φ (***)^
VPA	19.3 (10.7)	16.6 (14.5)	20.4 (11.0) ^φ (***)^
MVPA	62.3 (21.7)	54.1 (32.2)	65.5 (22.3) ^φ (***)^

Notes: PA = Physical Activity; M = Mean; SD = Standard Deviation; Min/day = Minutes Per Day; LPA = Light PA; MPA = Moderate PA; VPA = Vigorous PA; MVPA = Moderate to Vigorous PA; ^φ^ Significant difference (*p* < 0.05) between Weekend and Weekday; *** *p* < 0.001.

**Table 3:** PA by race, gender, grade, and weight status for whole week, weekend, and weekday.

Study Variables	MVPA (Min/day) M (SD)			≥60 Min MVPA % ^δ^	
	Total Week	Weekend	Weekday ^γ^	All Days	5 Days
Overall	62.3 (21.7)	54.1 (32.2)	65.5 (22.3) ^φ^^(***)^	9.1%	34.8%
Race ^α^					
Tibetan	67.4 (22.7)	63.7 (34.4)	68.6 (23.7) ^φ^^(**)^	13.5%	41.5%
Han	57.2 (19.5) ^a^^(***)^	44.4(26.6) ^a^^(***)^	62.3 (20.4) ^a^^(**)^^φ^^(***)^	4.6% ^a^^(**)^	27.9% ^a^^(**)^
Gender ^α^					
Male	71.5 (22.3)	60.7 (35.1)	75.6 (22.5) ^φ^^(***)^	13.8%	51.3%
Female	53.5 (17.0) ^b^^(***)^	47.8 (27.8) ^b^^(***)^	55.7 (17.2) ^b^^(***)^^φ^^(***)^	4.5% ^b^^(**)^	18.8% ^b^^(***)^
Grade by School Level ^β^					
PS	61.5 (21.8)	53.1 (30.4)	64.5 (22.0) ^φ^^(***)^	10.1%	35.3%
JHS	63.5 (22.8)	50.2 (30.4)	68.8 (24.3) ^φ^^(***)^	9.7%	37.9%
SHS	61.9 (20.4)	60.4 (36.0)	62.4 (19.4)	7.1%	30.1%
Weight Status ^α^					
Non-OW	62.3 (22.0)	54.1 (32.6)	65.5 (22.6) ^φ^^(***)^	9.0%	33.7%
OW/OB	62.6 (19.5)	54.4 (29.2)	65.7 (20.3) ^φ^^(**)^	9.8%	43.9%

Notes: MVPA = Moderate to Vigorous Physical Activity; M = Mean; SD = Standard Deviation; PS = Primary School; JHS = Junior High School; SHS = Senior High School; Non-OW = Non-overweight; OW/OB = Overweight/Obesity; ^α^ The Wilcoxon Two-Sample Test was used to test inter-group differences in MVPA with respect to ethnicity, gender and body weight status groups; ^β^ The Steel–Dwass Test was used to test the differences in MVPA between grade groups; ^γ^ The Wilcoxon Matched-Pairs Signed Ranks Test was used to test the difference in MVPA between weekends and weekdays; ^φ^ Significant difference between weekends and weekdays; ^δ^ Percentage values of inter-group differences was test by chi-square test; ^a^ Significant difference between Male and Female; ^b^ Significant difference between Non-OW and OW/OB; * *p* < 0.05, ** *p* < 0.01, *** *p* < 0.001.
